# Supplementary material for: Precision cutaneous stimulation in freely moving mice
Source: eLife. 2026 Mar 12;14:RP106033. doi: 10.7554/eLife.106033 (PMC12981839; doi:10.7554/eLife.106033)
Supplement: Supplementary file 1. — This table details the optical components for the assembly of the system. [file elife-106033-supp1.docx]

**Supplementary File 1. Optics**. This table details the optical components for the assembly of the system.

| **Optics** | | | |
| --- | --- | --- | --- |
| Description | Part reference | Information | Quantity |
| Diode laser (blue light), 473 nm, 100 mW | 06-01 MLD, Cobalt | Mounted on custom heatsink | 1 |
| Diode laser (infrared, IR), 785 nm | RLM785TA-1500, SLOC | Mounted on heatsink | 1 |
| Diode laser (blue light) alignment mirrors | BB1-E02, Thorlabs | M1 and M2 in Fig. 2 | 2 |
| Near-IR hot mirror | FM201, Thorlabs | Minimized IR laser light imaged on the camera | 2 |
| Mirror mounts | POLARIS-K1-H, Thorlabs | Mounts for M1 and M2 | 2 |
| Optical beam shutter | SH05RM, Thorlabs | Controlled IR beam path | 1 |
| Lens, 30 mm focal length | AC254-030-A, Thorlabs | L1 in Fig. 2 | 1 |
| Solenoid controller | KSC101, Thorlabs | Controlled optical beam shutter | 1 |
| Lens, 150 mm focal length | AC254-150-1-ML, Thorlabs | L2 in Fig. 2 | 1 |
| Near-IR lens, 750 mm focal length | AC508-750-B, Thorlabs | L4 in Fig. 2 | 1 |
| Lens, 500 mm focal length | AC254-500-A-ML, Thorlabs | L3 in Fig. 2 | 1 |
| IR laser alignment mirrors | BB1-E03, Thorlabs | M3 and M4 in Fig. 2 | 2 |
| Non-rotating adjustable lens housing | SM1NR1, Thorlabs | Housing for L1 | 1 |
| Mirror mounts | KCB1C/M, Thorlabs | Mounts for M3 and M4 | 2 |
| Lens mount | LMR1/M, Thorlabs | Mounts L2 and L3 | 1 |
| ND filter, OD 0.3 | NE30A-B, Thorlabs | Used for IR laser measurements | 1 |
| Neutral density filter wheel | SCFW6, Thorlabs | F in Fig. 2 | 1 |
| Dichroic mirror | DMLP567R, Thorlabs | 25 mm x 36 mm DM in Fig. 2 | 1 |
| ND filter, OD 0.5 | NE505B, Thorlabs | Housed in SCFW6 for 32% transmittance or with 1x NE510B for 3.2% transmittance | 2 |
| DM mount | CM1-DCH/M, Thorlabs | Houses DM | 1 |
| ND filter, OD 1.0 | NE510B, Thorlabs | Housed in SCFW6 for 10% transmittance or with NE505B for 3.2% transmittance | 2 |
| End cap | SM1CP2, Thorlabs | Blocks one port of CM1-DCH/M | 1 |
| ND filter, OD 1.3 | NE513B, Thorlabs | Housed in SCFW6 for 5% transmittance | 1 |
| Mirror galvanometer system | GVSM002/M, Thorlabs | *x*-axis and *y*-axis GM, mount, servos and power supply | 1 |
| ND filter, OD 2.0 | NE520B, Thorlabs | Housed in SCFW6 for 1% transmittance | 1 |
| Galvanometer mirror mount | GCM002/M, Thorlabs | Housed GM | 1 |
| Glass stimulation platform | Custom | Borosilicate glass, 550 mm x 550 mm x 5 mm | 1 |
