# Supplementary material for: Precision cutaneous stimulation in freely moving mice
Source: eLife. 2026 Mar 12;14:RP106033. doi: 10.7554/eLife.106033 (PMC12981839; doi:10.7554/eLife.106033)
Supplement: Supplementary file 2. — This table details the parts for mounting optics in the system. [file elife-106033-supp2.docx]

**Supplementary File 2. Mounting components**. This table details the parts for mounting optics in the system.

| **Mounting components** | | | |
| --- | --- | --- | --- |
| Description | Part reference | Information | Quantity |
| Aluminum breadboard | MB7575/M, Thorlabs | See Fig. 2 | 1 |
| Optical 95 mm construction rail | XT95-1500, Thorlabs | Glass platform is attached (see Fig. 2) | 1 |
| Optical rail end plate | XT95EC1, Thorlabs | Attached to the top of the optical rail | 1 |
| Optical rail mounting plate | XT95P3, Thorlabs | Mounts optical rail | 1 |
| Optical 25 mm construction rail | XT95-500, Thorlabs | Structure that held the glass platform (see Fig. 2) | 1 |
| Corner cube for 25 mm rail | RM1G, Thorlabs | Structure that held the glass platform (see Fig. 2) | 4 |
| Aluminum 25 mm construction rail | XE25L500/M, Thorlabs | Mounted around the glass platform for stability | 5 |
| Right-angle bracket | AB09H, Thorlabs | Mounts aluminum rails to construction rails holding glass platform | 5 |
| Post, 150 mm | TR150/M, Thorlabs | Mounted Basler camera | 1 |
| Posts, 30 mm | TR30/M, Thorlabs | Mounted M1, F, L2, L3, M3, M4, DM | 7 |
| Post, 20 mm | TR20/M, Thorlabs | Mounted M2 | 1 |
| Post holder, 150 mm | PH150/M, Thorlabs | Holder for TR150/M post | 1 |
| Post holders, 30 mm | PH30/M, Thorlabs | Holders for TR30/M posts | 7 |
| Post holder, 20 mm | PH20/M, Thorlabs | Holder for TR20/M post | 1 |
| Clamping fork | CF125C/M, Thorlabs | Clamps optomechanical component assembly to larger breadboard (see Fig. 2) | 1 |
