# Supplementary material for: Precision cutaneous stimulation in freely moving mice
Source: eLife. 2026 Mar 12;14:RP106033. doi: 10.7554/eLife.106033 (PMC12981839; doi:10.7554/eLife.106033)
Supplement: Supplementary file 3. — This table details the parts for acquisition and control. [file elife-106033-supp3.docx]

**Supplementary File 3. Acquisition and control components**. This table details the parts for acquisition and control.

| **Acquisition and control components** | | | |
| --- | --- | --- | --- |
| Description | Part reference | Information | Quantity |
| USB 3.0 Camera | acA1920-40um, Basler | Acquired at 1920 x 1200 pixels, 30 fps | 1 |
| Camera lens | LM6HC, Kowa | Attached to Basler camera | 1 |
| IR LED panels | 12V 850 nm LEDs, SMD3528-300-IR, 4.8 W/m | Illuminated either side of the glass platform | 2 |
| LED power supply | 12V DC power | Powers LED panels | 2 |
| Primary computer (C1) | Custom | Controlled pose estimation, targets GM, and acquires data | 1 |
| Secondary computer (C2) | Custom | Controlled laser software and audio | 1 |
| Multifunction I/O device | USB-6211, National Instruments | Interfaces between PC1 and PC2, GM, lasers, Arduino UNO | 2 |
| Arduino | Arduino UNO | Controlled lasers and reward ports | 2 |
